# Infant Brain Atlases from Neonates to 1- and 2-Year-Olds

**DOI:** 10.1371/journal.pone.0018746

**Published:** 2011-04-14

**Authors:** Feng Shi, Pew-Thian Yap, Guorong Wu, Hongjun Jia, John H. Gilmore, Weili Lin, Dinggang Shen

**Affiliations:** 1 IDEA Lab, University of North Carolina at Chapel Hill, Chapel Hill, North Carolina, United States of America; 2 Department of Psychiatry, University of North Carolina at Chapel Hill, Chapel Hill, North Carolina, United States of America; 3 MRI Lab, Department of Radiology and BRIC, University of North Carolina at Chapel Hill, Chapel Hill, North Carolina, United States of America; Tokyo Medical and Dental University, Japan

## Abstract

**Background:**

Studies for infants are usually hindered by the insufficient image contrast, especially for neonates. Prior knowledge, in the form of atlas, can provide additional guidance for the data processing such as spatial normalization, label propagation, and tissue segmentation. Although it is highly desired, there is currently no such infant atlas which caters for all these applications. The reason may be largely due to the dramatic early brain development, image processing difficulties, and the need of a large sample size.

**Methodology:**

To this end, after several years of subject recruitment and data acquisition, we have collected a unique longitudinal dataset, involving 95 normal infants (56 males and 39 females) with MRI scanned at 3 ages, i.e., neonate, 1-year-old, and 2-year-old. State-of-the-art MR image segmentation and registration techniques were employed, to construct which include the templates (grayscale average images), tissue probability maps (TPMs), and brain parcellation maps (i.e., meaningful anatomical regions of interest) for each age group. In addition, the longitudinal correspondences between age-specific atlases were also obtained. Experiments of typical infant applications validated that the proposed atlas outperformed other atlases and is hence very useful for infant-related studies.

**Conclusions:**

We expect that the proposed infant 0–1–2 brain atlases would be significantly conducive to structural and functional studies of the infant brains. These atlases are publicly available in our website, http://bric.unc.edu/ideagroup/free-softwares/.

## Introduction

An atlas usually refers to a map with spatial recording of relevant information. Brain atlases, embedding knowledge of structural and functional properties of neuroanatomical sites, are widely used in computational neuroanatomy for pedagogical purposes, surgical planning, disease diagnosis, and medical image analysis [1], [2], [3]. An atlas, in its different forms, can be used as a reference for normalization of a group of individuals, a probability map for defining tissue prior distribution, or a spatial map for brain parcellation. Brain atlases were initially collections of detailed drawings of brain structures by anatomists according to the autopsy of individual subjects (see [4] for review). These paper atlases have facilitated a great measure of success in understanding the underlying anatomy of human brains. With the advancement of computing and medical imaging technologies, digital brain atlases constructed for different imaging modalities are increasingly more common and provide more precise delineation of brain structures and allow automatic processing of large datasets with minimal human intervention.

Many existing brain atlases are based on a single subject or a limited number of individuals, e.g., the Brodmann atlas [5] and the Talairach & Tournoux atlas [6]. Atlases as such cannot guarantee capturing of subject-independent information which caters for a broader range of human brains, and can thus cause problem when used to propagate on-atlas information to any other subject due to confounding anatomical variability [7]. In response to this, population-based atlases were introduced, e.g., the MNI305 [8] and ICBM152 brain atlases [9], which were obtained by averaging the anatomical magnetic resonance (MR) images of 305 and 152 adult brain images, respectively. Besides normal brain atlases, disease-specific atlases as well as genetic atlases of humans were also widely studied [10], [11], [12], [13], [14], [15], [16].

Although numerous human brain atlases have been produced, they are mostly developed for adults. Infant atlases, however, are not well developed. Recent studies suggested that using adult or even pediatric atlases may compromise accuracy in analyzing infant brain images [11]. The degraded performance stems from the fact that dynamic and significant growth processes occur in the first years of life; thus, an atlas not created for infants simply fails to reflect their anatomy. Three major difficulties associated with changes due to development, MR imaging inconsistency, and cohort size confound the efforts of constructing infant atlases. *First*, fast development of the infant brain demands dedicated infant atlases constructed for specific age group (i.e., time-point), e.g., neonates, 1-year-olds, and 2-year-olds. As reported in [17], MR imaging indicates that the neonatal brain is only half the volume of adult brain, and grows to about 90% adult brain volume at the end of the second year. Similarly, the white matter (WM) myelination process is also associated with early brain development. Most WM is unmyelinated in neonates. Myelination progresses in the brain from central to peripheral, from inferior to superior, and from posterior to anterior. This process continues in the 1-year-old brain with adult-like pattern occurring in the 2-year-old brain [18]. Due to this dynamic change in the first years of life, atlases representing neonates, 1-year-olds, and 2-year-olds should characterize specific anatomical patterns. Therefore, atlases constructed for each of the three infant stages are highly desired. By doing so, more age-related anatomical characteristics can be preserved in each stage. *Second*, many studies are obstructed by the quality insufficiency of infant MR images, which is even more severe in the case of neonatal images [19], [20]. Specifically, due to the small brain size and developing tissue properties, the quality of infant images is typically poor with insufficient spatial resolution, low tissue contrast, and ambiguous tissue intensity distribution, which confound subsequent operations such as tissue segmentation and image registration. Dedicated infant segmentation methods need to be employed to handle image quality problems and to provide reliable results for ensuring the success of subsequent operations. *Third*, a large sample size is desired for constructing atlases. However, obtaining infant MRI data can be difficult [11]. Acquisition of images of infants requires additional effort in ensuring that the infant remains still throughout scanning – something which can be achieved easily by adults, but much less easily by infants. Moreover, the availability of longitudinal follow-ups is also highly dependent on the cooperation of the infants and their parents. All these factors hinder the collection of a substantial number of images that are required for building a reliable atlas.

**Figure 1:**
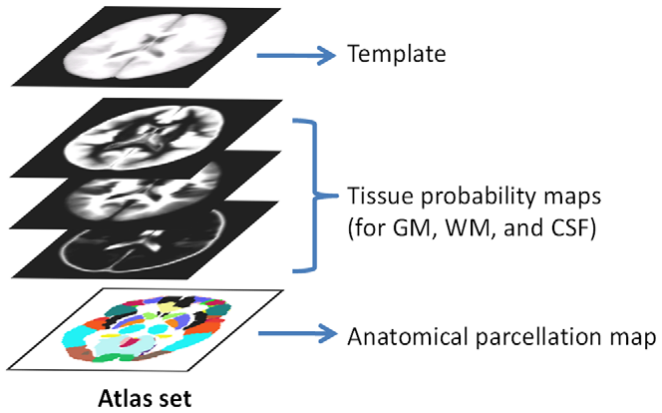
A sample atlas and its components.

Based on the typical applications, an atlas should comprise the following major components (see Fig. 1): (1) a template (i.e., grayscale average image), which serves as the registration reference for spatial normalization of a population of images; (2) a set of tissue probability maps (TPMs) of gray matter (GM), white matter (WM) and cerebrospinal fluid (CSF) for guiding segmentation; (3) an anatomical parcellation map for structural labeling. An atlas as such is highly desired for infant related studies.In the upper panel of Table 1, we list a number of recently published infant studies, in which template, TPMs, and anatomical parcellation map were constructed to facilitate their subsequent segmentation or structural labeling process [18], [19], [20], [21], [22], [23], [24], [25]. In most studies, the components of atlases were obtained by directly averaging a number of presegmented and aligned infant images. Some others, like Xue et al., simulated the TPMs by using the initial segmentation of the to-be-segmented image by *k*-means clustering and Gaussian blurring [20]. Gilmore et al. proposed a neonatal brain parcellation map, where 16 cortical regions, 20 subcortical regions, brainstem, and cerebellum were defined by anatomical experts [18]. Studies dedicated to infant atlas construction and their atlases currently publicly available were listed in the lower panel of Table 1 [11], [12], [26], [27]. Kazemi et al. provided an infant template [11]. Kuklisova-Murgasova et al. presented a 4D atlas particularly for premature neonates, by using 142 subjects with average gestation age of 29.4±2.7 weeks at birth [26]. A 1-year-old atlas was constructed in [12], with template and TPMs available. Gousias et al. non-rigidly warped 30 manual parcellation maps of 30 normal adult brains (each containing 83 anatomical regions) onto each of 33 2-year-old subjects, then fused the 30 warped label maps into a final map for each 2-year-old subject [27]. As clearly indicated in Table 1, few atlases targeting normal newborns are publicly available, and they generally have limited features to meet various image processing needs. Specifically, in the context of the three main atlas applications in infant studies – spatial normalization using templates, guiding tissue segmentation using TPMs, and structural labeling using an anatomical parcellation map, we observed form the atlases provided in the above studies that: (1) there are no available TPMs that can be used for guiding tissue segmentation of normal neonatal subjects; (2) also missing are the neonatal anatomical parcellation maps, which are very important for automated delineation of regions of interest (ROIs) in fMRI or DTI studies; (3) if atlases are jointly used in the studies involving different age groups, such as combing the neonate [11], 1-year-old [12], and 2-year-old atlases [27], the consistency in the obtained results may be questionable, because atlases were constructed from different subjects and groups using different segmentation and registration methods.

**Table 1:** Summary of recently published atlas-related infant brain studies.

	N	Age at MRI	MR Field Strength	Template	Tissue Probability Maps	Anatomical Parcellation Map	Public Availability
***Studies that constructed templates, TPMs, and parcellation maps***							
Prastawa et al., 2005	3	Neonate	3T	Yes	Yes	-	-
Weisenfeld et al., 2006a	13	Neonate (GA 42 weeks)	1.5T	Yes	Yes	-	-
Weisenfeld et al., 2006b	20	Neonate (GA 42 weeks)	1.5T	Yes	Yes	-	-
Xue et al., 2007	25	Neonate (GA 35 weeks)	3T	Yes	Yes	-	-
Song et al., 2007	9	Neonate (PA<10 days)	-	Yes	Yes	-	-
Gilmore et al., 2007	-	Neonate	-	-	-	38 ROIs	-
Weisenfeld et al., 2009	15	Neonate (GA 40 weeks)	1.5T	Yes	Yes	-	-
Shi et al., 2010b	68	Neonate (GA 41 weeks)	3T	Yes	Yes		
***Studies that established infant atlases for public use***							
Kazemi et al., 2007	7	Neonate (GA 39-42 weeks)	1.5T and 3T	Yes	-	-	http://www.u-picardie.fr/labo/GRAMFC
Kuklisova-Murgasova et al., 2010	142	Premature neonate (GA 29-48 weeks)	3T	Yes	Yes	-	www.brain-development.org
Altaye et al., 2008	76	1-year-old	3T	Yes	Yes	-	https://irc.cchmc.org/software/infant.php
Gousias et al., 2008	33	2-year-old	1T	Yes	-	83 ROIs	http://www.doc.ic.ac.uk/~dr/brain-development/

Note: “Yes” means the item was generated in the study. “-“ means not available. GA means gestational age. PA means postnatal age. ROI means region of interest.

In this paper, we aim to construct a set of dedicated infant atlases for neonates, 1-year-olds, and 2-year-olds, referred to as infant 0–1–2 atlases. First, a total number of 95 subjects with complete 0–1–2 longitudinal scans were collected. Second, we apply state-of-the-art infant longitudinal segmentation [28] and groupwise registration techniques [29] for constructing the brain atlases. Third, an atlas is constructed for each of the three age groups, together with the relevant longitudinal correspondences. Our approach is detailed in the Method section. We then describe experiments to evaluate the performance of the proposed atlases in typical infant studies related applications. The Discussion section highlights the novelty of the proposed method and some possible future research directions. Finally, the Conclusion section concludes the paper.

## Materials and Methods

### 2.1 Subjects and MRI Acquisition

Subjects used in this paper were part of a large study of early brain development in normal children [18]. The experimental protocols were approved by the institutional review board of the University of North Carolina (UNC) School of Medicine. The parents were recruited during the second trimester of pregnancy from the UNC hospitals and written informed consent forms were obtained from all the parents. The presence of abnormalities on fetal ultrasound, or major medical or psychotic illness in the mother, was taken as exclusion criteria. The infants were free of congenital anomalies, metabolic disease, and focal lesions. None of the subjects was sedated for MRI. Before the subjects were imaged, they were fed, swaddled, and fitted with ear protection.

Images were acquired on a Siemens head-only 3T scanner (Allegra, Siemens Medical System, Erlangen, Germany) with a circular polarized head coil. For T1-weighted images, 160 sagittal slices were obtained by using the three-dimensional magnetization-prepared rapid gradient echo (MPRAGE) sequence: TR = 1900 ms, TE = 4.38 ms, inversion time = 1100 ms, Flip Angle = 7°, and resolution = 1×1×1 mm^3^. For T2-weighted images, 70 transverse slices were acquired with turbo spin-echo (TSE) sequences: TR = 7380 ms, TE = 119 ms, Flip Angle = 150°, and resolution = 1.25×1.25×1.95 mm^3^. Data were collected longitudinally at 3 age groups: neonates, 1-year-olds, and 2-year-olds. Data with motion artifacts was discarded and a rescan was made when possible. Finally, complete 0–1–2 data of 95 normal infants was acquired. The demographic information was summarized in Table 2. Gestational ages were between 38.7 and 46.4 weeks at the first dates of examination. The variation of age at MRI for each scan is relatively small and the population can be divided in age groups concentrated around 0, 1, and 2 years of age.

**Table 2:** Demographic information of the normal infants used in this study.

Scan	N	Gender	Age at Birth (weeks)	Age at MRI (weeks)	Group
**First**	95	56 males/39 females	37.9±1.8 (33.4–42.1)	41.5±1.7 (38.7–46.4)	Neonate
**Second**				94.2±3.4 (87.9–109.1)	1-year-old
**Third**				146.2±4.9 (131.4–163.4)	2-year-old

Note: GA means gestational age.

### 2.2 Image preprocessing

Before further operation, all images were preprocessed using a standard procedure. Non-brain tissues such as skull and dura were stripped with Brain Surface Extractor (BSE) [30], followed by manual editing with ITK-SNAP software [31] to ensure accurate skull removal. Bias correction was performed on all images with nonparametric nonuniform intensity normalization (N3) method [32] to reduce the impact of intensity inhomogeneity and thus improve the performance of the subsequent tissue segmentation. T2-weighted images were resampled to have a resolution of 1×1×1 mm^3^. Note that one image modality with better tissue contrast was selected for each age group for delineation of anatomical patterns: T2 for neonates, and T1 for 1- and 2-year-olds [28].

### 2.3 Data process and atlas construction

**Figure 2:**
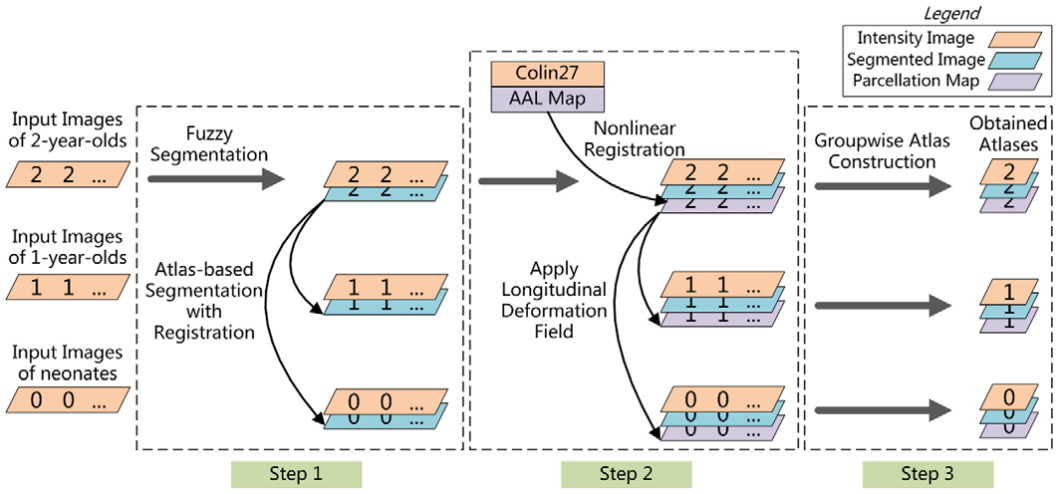
The infant 0–1–2 atlas construction framework. From left to right, three main steps are involved in constructing the atlases: longitudinal tissue segmentation (step 1), anatomical labeling (step 2), and unbiased groupwise atlas construction (step 3). Note that the cross-sectional and longitudinal registrations in the three steps were performed on the segmented images, since the intensity profile would change dramatically due to the myelination and maturation.

To build the infant 0–1–2 atlases, the images need to be segmented, registered to a common space, and averaged to generate the atlases, representing subject-independent population information. The key to atlas construction involves performing accurate tissue segmentation to identify tissue structures and robust registration to determine the anatomical correspondences across age groups and subjects. Specifically, we perform three steps, i.e., longitudinal tissue segmentation, anatomical labeling, and unbiased groupwise atlas construction, for constructing the atlases as illustrated in Fig. 2.

#### 2.3.1 Step 1: Longitudinal Tissue Segmentation

To build an atlas, a given population of brain images needs to be segmented. The quality of the final atlas is directly related to the segmentation accuracy. However, manual segmentation is tedious, time-consuming, and may lack reproducibility. As suggested in many studies [12], [19], [24], [28], automated segmentation of neonatal brain images remains a challenging problem due to poor image quality (low spatial resolution and tissue contrast) and high within-tissue intensity variability. However, it is generally agreed that segmentation of 2-year-old images is relatively easy since they are beginning to exhibit early-adult-like structural patterns [28]. It is also noticed that major brain structures remain similar during postnatal development [33]. For instance, the major cortical gyrification has developed sufficiently during gestation in the third trimester, and the cortical convolution patterns remain similar even after normal birth. For these reasons, in our previous work, we have used the warped TPMs of 2-year-olds as subject-specific tissue probabilistic priors for guiding segmentation of their respective neonatal images [28]. Compared with priors gathered from images of different individuals, the subject-specific prior exhibits smaller anatomical variability with the to-be-segmented image, and thus provides more accurate and longitudinally consistent segmentation results.

Specifically, as shown in Step 1 of Fig. 2, the 2-year-old images were first segmented by an adaptive fuzzy c-means (AFCM) algorithm [34]. The resulting GM, WM and CSF TPMs were then used as subject-specific priors for guiding segmentation of the 1-year-old and neonatal images. A joint registration-segmentation approach [28] was adopted: (1) a registration step for aligning the 2-year-old image onto the early time-point image based on their segmented images (Note that the initial registration was based on intensity images), and (2) a segmentation step for refining the segmentation result of the early time-point image based on the aligned TPMs. Segmentation is implemented utilizing TPMs in conjunction with the subject's intensity model. These two steps are iterated to refine the anatomical correspondences between the 2-year-old image and the neonatal/1-year-old image, and to improve the segmentation accuracy of the neonatal/1-year-old image. The final outcome is the segmentation results at all three age groups, as well as the longitudinal deformation fields relating all images. In particular, in the segmentation step, multiple Gaussians are employed to model the intensity distribution of each brain tissue. For example, although myelinated and unmyelinated WM have very different intensity profiles, they can be jointly modeled by multiple Gaussians with help of tissue priors obtained from 2-year-old image. The final segmentation results of all images were visually inspected by a trained rater, to verify the segmentation quality and remove possible artifacts or errors generated by the automated segmentation algorithm.

#### 2.3.2 Step 2: Anatomical Labeling

An anatomical parcellation divides the brain into multiple non-overlapping volumes of interest (VOIs). Usually, the parcellation map of a single-subject atlas is obtained by manual delineation, such as the Brodmann map which is based on cortical cytoarchitectonic organisation of neurons [5]. Alternatively, the Automated Anatomical Labeling (AAL) map is based on anatomical localization such as gyrus and sulcus [35]. There are generally two ways to automatically generate a parcellation map for a group of subjects: (1) Direct warping way, which directly warp an existing atlas to the mean image of the population for obtaining parcellation; (2) Indirect fusion way, which warps an existing atlas to each subject of the population and then fuses the warped multiple parcellation maps into a final result. A fusion-based approach as such has been proven to be more efficient than the direct warping approach, and could result in a better-quality parcellation map [13], [36]. In this paper, we use the indirect fusion approach to propagate the AAL map to our infant subjects.

The AAL map was originally defined on the Montreal Neurological Institute (MNI) single subject brain MR image [35]. This brain image, Colin27, was created by averaging 27 registered scans of a single subject, Colin Holmes [37]. 45 anatomical VOIs in each hemisphere were defined based on anatomical characteristics, i.e., using main sulci as landmarks. To reliably determine anatomical correspondence between the Colin27 brain and the infants, we propose to use the 2-year-old image of the same subject as intermediate image for guiding the registration. Specifically, we first warp the Colin27 brain to each 2-year-old image in the population, and then propagate the warped parcellation map longitudinally to its corresponding early-time image. By using the 2-year-old image as a bridge image, we can avoid the direct registration of adult AAL brain with neonatal brain image (0-year-old image) and thus significantly reduce the possible registration error. Note that the strategy of using the intermediate images as bridge for guiding registration has been recently employed as an effective technique in various groupwise registration methods, in which a registration pathway containing multiple images as intermediate bridges is determined for progressively registering one image to another image [38], [39]. By using this approach, the parcellation maps of all subjects in each group were obtained and then fused to generate the final parcellation map by voxel-wise majority voting for the particular group. The resulting infant AAL map would facilitate infant studies, such as region of interest (ROI) localization in fMRI studies and volume changes of specific anatomical regions.

Specifically, as shown in Step 2 of Fig. 2, the anatomical correspondences between Colin27 brain to the early-adult-like 2-year-old images were estimated by a hierarchical nonlinear deformable registration algorithm, namely HAMMER [40], [41], based on their segmented images. Subvoxel registration accuracy (i.e., average 0.63 mm) could be achieved [41]. The resulting deformation fields were then employed to warp the AAL map to each of the 2-year-old images. The warped parcellation maps were then propagated to the neonatal or 1-year-old image of the same subject, with the longitudinal deformation fields obtained in Step 1. Finally, we obtain a set of images, including intensity images, TPMs, and anatomical parcellation maps for each subject at each age group.

#### 2.3.3 Step 3: Unbiased Groupwise Atlas Construction

To generate an atlas from a population, one subject is usually selected as a reference template, to which all images are registered. This explicit template selection may bias the subsequent data analysis, and can be avoided by recent advancement in registration techniques such as groupwise registration [29], [42], [43]. Therefore, we employ a recently developed feature-based groupwise registration algorithm [29] to align the subjects in each age group to their age-specific common space.

As shown in Step 3 of Fig. 2, for each age group, the individual images of the 95 subjects were simultaneously registered to the common space. Briefly, all images were first roughly aligned together by using affine transformation. Then nonlinear groupwise registration [29] was employed. For each voxel, image features consisting of image intensity, edge type, and geometric moment invariants on three tissue types (WM, GM, and CSF) were first computed. Then only the driving voxels, i.e., the anatomically distinctive voxels (e.g., in gyral crowns and sulcal roots) were allowed to participate in identifying the correspondences. The warping of other non-driving voxels was guided by the deformation given by these driving voxels. Specifically, for each driving voxel, we searched in the neighborhood of the subject image for a number of candidate matching points based on feature similarity. The driving voxel under consideration was then moved to the mean location of all candidate points. Thin-plate splines were adopted to interpolate the sparse correspondences to obtain a dense transformation field for warping each subject to the common space. By repeating these steps, i.e., correspondence detection and dense deformation estimation, we are able to simultaneously register all subject to the hidden common space and also the mean image.

After groupwise registration, the atlases and TPMs were obtained by averaging the relevant aligned images. Anatomical parcellation map was obtained by performing majority voting on the aligned parcellation maps. The proposed infant 0–1–2 atlases constructed using the three above-mentioned steps were evaluated in the experimental section below.

Longitudinal correspondences established across three age groups are very useful, i.e., to consistently transform subjects from one common space to another common space of the 3 ages. To construct this type of longitudinal correspondences, we first computed the deformation fields 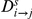 between any two ages (age *i* and 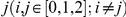) for each subject 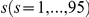, as described in step 1. Then, for each age *i* of each subject *s* we obtained its deformation field 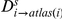 to its respective common space of age *i*, *atlas*(*i*), as described in step 3. Thus, by composing the deformations 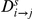 and 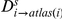, 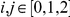}, we could get the longitudinal deformations/correspondences for each subject *s* in the common spaces of 3 ages. We then further averaged these longitudinal deformations/correspondences from all 95 subjects, to obtain the final averaged longitudinal correspondences that connected the atlases of these 3 ages.

## Results

### 3.1 Overview of the proposed infant 0–1–2 atlases

**Figure 3:**
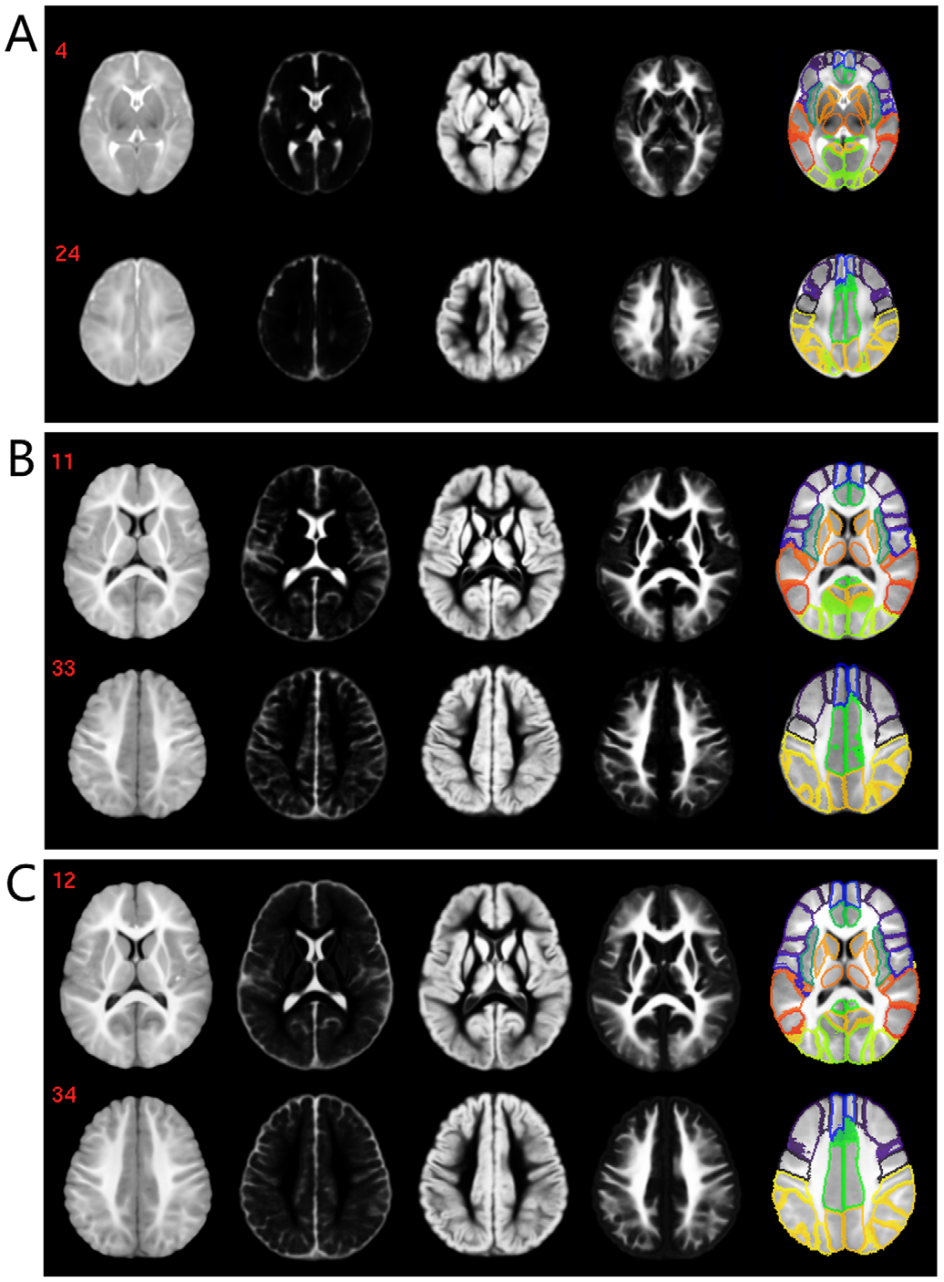
Illustration of the infant 0–1–2 atlases. (A–C) are for neonates, 1-year-olds, and 2-year-olds, respectively. Brain sizes of the three age groups are shown in proportion. T2 images were used for neonates, and T1 for 1- and 2-year-olds. In each panel, from left to right are the template, three TPMs for CSF, GM, WM, and anatomical parcellation map; from top to bottom, two representative slices are shown. Values on the upper left of each slice indicate the stereotaxic z coordinate in millimeters. Note that region boundaries are shown in the parcellation maps for better visualization.

The atlases constructed for neonates, 1-year-olds, and 2-year-olds are shown in Fig. 3 (A–C), respectively. From top to bottom in each panel are the template, TPMs (for CSF, GM, and WM), and anatomical parcellation map, respectively. The reference coordinate space of the proposed atlases was represented by 3D images with dimensions of 181×217×180 and resolution of 1×1×1 mm^3^. Origin was set at slice 90 in x, 126 in y, and 72 in z as the appearance of anterior commissure. The orientation of anterior-posterior commissure is parallel with the anterior-posterior axis of the image.

**Figure 4:**
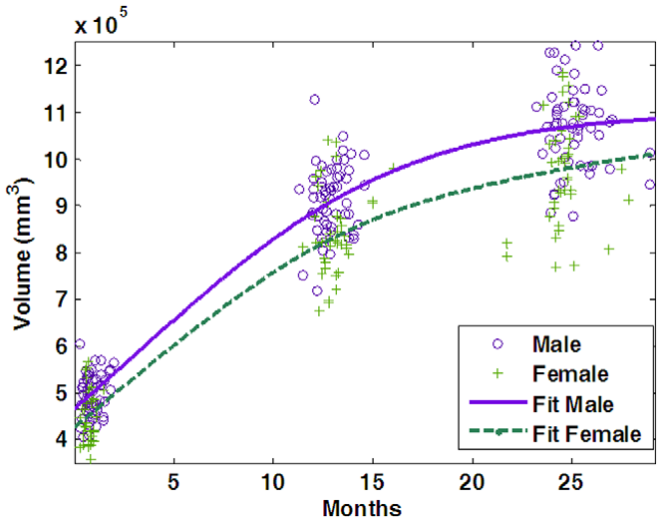
Scatter plot of cerebral volumes for the 95 subjects. The fitted curves show the development trend of the male and female subjects, respectively.

Fig. 4 shows the scatter plot of the cerebral volumes of the 56 males and 39 females in our dataset for each age group. It can be observed that the brain grows rapidly from neonates to 1-year-olds, and then slows down from 1-year-olds to 2-year-olds. Meanwhile, males generally have larger cerebral volume than female. The finding is in agreement with previous study [17].

### 3.2 Evaluation Based on Typical Applications

**Figure 5:**
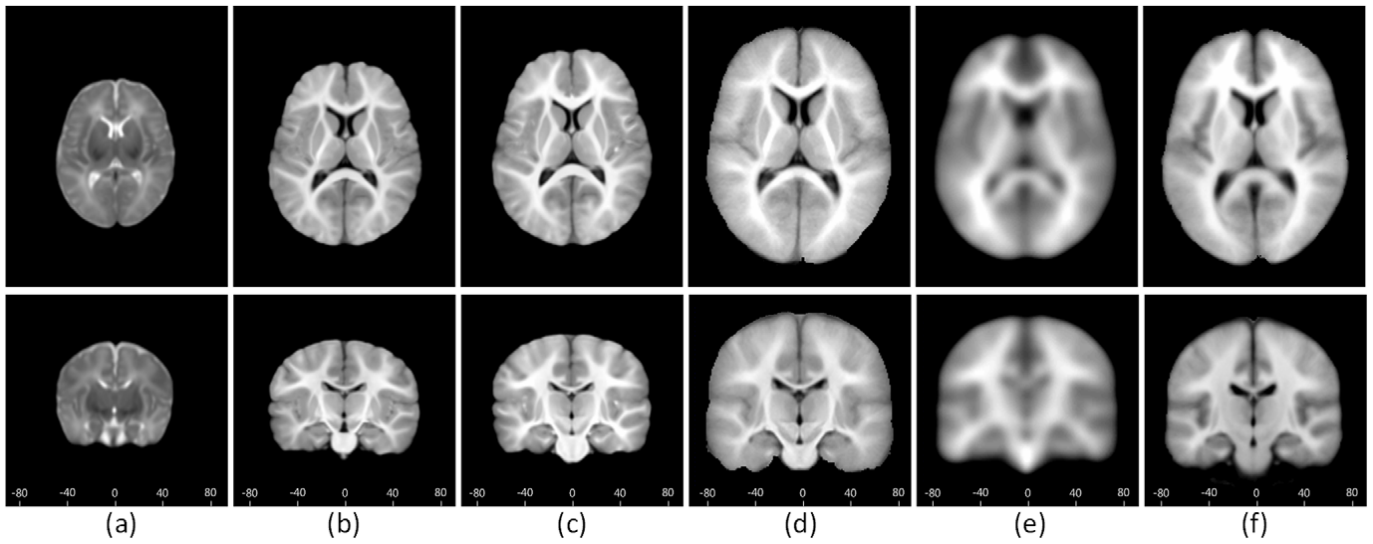
Examples of templates of the proposed atlases and 3 other publicly available atlases. (a-c) our proposed neonate, 1-year-old, and 2-year-old atlases; (d) CCHMC-Infant atlas; (e) CCHMC-young atlas; (f) ICBM-Adult atlas. An axial and a coronal slices are shown for each atlas Values on the bottom of each slice indicate the stereotaxic y coordinate in millimeters. Note that the substantial fuzziness can be observed in cortical regions, when comparing (d–e) with our proposed atlases (a–c).

For evaluation, we randomly selected 75 out of 95 subjects to construct the template, TPMs, and anatomical segmentations. The remaining 20 subjects, not involved in the construction process, were used as test subjects. We further included three previously-published atlases as controls for performance comparison. The first atlas is a 1-year-old infant atlas (cited in Table 1), constructed from images of 76 infants with age ranging from 6 to 15 months, collected at the Cincinnati Children's Hospital Medical Center (CCHMC) (https://irc.cchmc.org/software/infant.php) [12]. Subject ages in this atlas match with ages of subjects in this study. We refer to this atlas as the CCHMC-Infant atlas. The second atlas is a pediatric atlas constructed from 67 young children with age ranging from 5 to 9.5 years, also collected at CCHMC, which we refer to as the CCHMC-Young atlas (https://irc.cchmc.org/software/pedbrain.php) [44]. The third is an adult atlas constructed from 152 adult subjects with age ranging from 18 to 44 years old, gathered in the ICBM project, which we refer to as the ICBM-Adult atlas (http://www.bic.mni.mcgill.ca/ServicesAtlases/) [45]. Typical image slices of the proposed atlases and the control atlases are shown in Fig. 5.

We demonstrate the advantages of the proposed atlases by showing that, first, in the same age range, they outperform the 1-year-old CCHMC-Infant atlas; second, as age-matched atlases, they outperform both young-children-atlas and adult-atlas when handling infant datasets; and third, as longitudinal atlases, they can achieve better consistency when processing images acquired longitudinally. Experiments were designed for evaluation of the proposed atlases in three applications, typical in infant related studies. *Experiment 1: Spatial Normalization.* Templates from atlases were used to normalize test subjects into a common space. After normalization, we evaluated the overlap of the warped brain structures across subjects. Better normalization will lead to a higher overlap value, indicating better representativeness of the atlas. *Experiment 2: Label Propagation*. Atlases were aligned to test subjects and the brain parcellation maps were then propagated to all three age groups of test subjects. We then compare the consistency of the warped parcellation maps from different atlases. *Experiment 3: Neonatal Segmentation.* 10 neonatal images were manually segmented to test the accuracy of automatic atlas-based segmentation by using 4 different atlases.

Registration between atlases and test subject images in the following experiments were performed using a technique [46] available in Statistical Parametric Mapping (SPM5) software package (http://www.fil.ion.ucl.ac.uk/spm/). Default parameters were used. In particular, the registration was performed by first using the “Normalise” module in SPM5 to conduct a 12-parameter affine transformation and then employing a linear combination of low-spatial-frequency discrete cosine transform (DCT) basis functions to represent deformations and conduct a non-rigid regsitration. The number of the basis functions is set to 7×8×7, resulting in 1176 parameters in three directions. To match the quality of the reference image (which is generally fuzzy), all input subject images were smoothed by convolving with an isotropic 8 mm FWHM Gaussian kernel, before registration. Global variability can be accommodated in this registration process, while subtle inter-subject variances were preserved, which makes it possible for the subsequent atlas performance comparison.

To measure the mean overlap between two segmentations, we employed the Dice ratio (DR) [47]. For two regions *A* and *B*, the Dice ratio is defined as 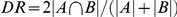. The numerator represents the number of voxels with the same label in both images, and the denominator represents the total sum of voxels in images *A* and *B.* The value of the DR ranges from 0 to 1, with both ends corresponding to the worst and the best agreements between labels of two regions, respectively.

#### 3.2.1 Spatial Normalization

**Figure 6:**
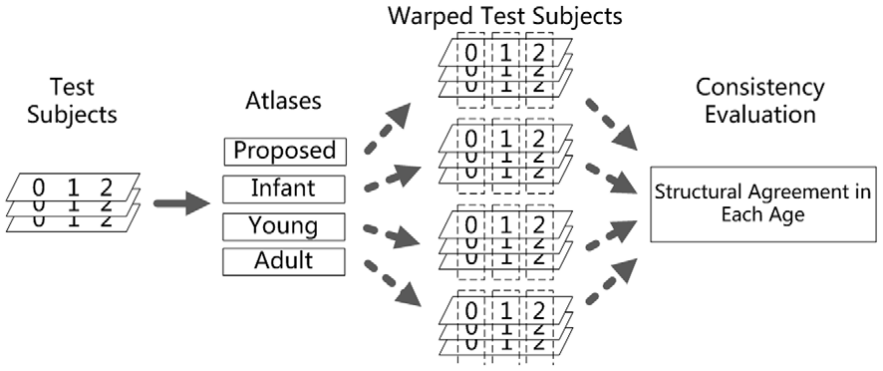
Flowchart for the spatial normalization experiment. Intensity images of subjects were aligned to the templates of atlases by using the normalise module in SPM5. Brain structures, i.e., the GM, WM, and CSF in segmented images, were compared with the population voted structure image for consistency evaluation.

Spatial normalization is widely used to allow a population of subjects to be transformed to a common space for subsequent statistical analysis. The selection of the template is essential for representing the structures of the population to achieve the spatial consistency of normalized subjects. In this experiment, the intensity images of 20 test subjects were normalized to the templates of the proposed age-matched atlas and the other 3 control atlases independently, by using the normalise module in SPM5. Schematic diagram is shown in Fig. 6. Brain structures, i.e., the GM, WM, and CSF in segmented images, were also aligned to the common space by using the result deformation fields. Since the ground truth for normalization is not available, we generate a mean image representing the structure of population by using a voxel-wise majority voting on the aligned segmented images. Brain structures of warped subjects were then compared with the population voted structure image, and the structural agreement was assessed by the *DR*.

**Figure 7:**
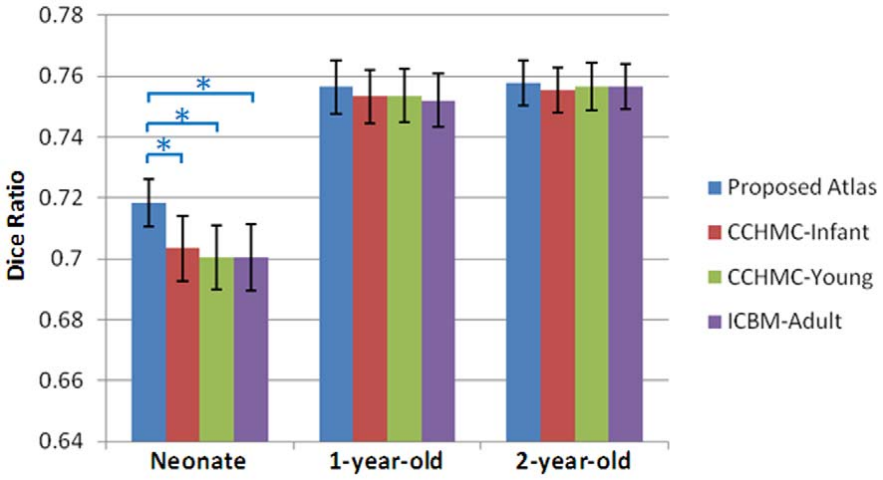
Dice ratios of structural consistency between the warped subjects and voted population for 4 atlases in 3 different age groups. “*” represents the significant difference between groups under comparison (p<0.05).

The mean and standard deviation of the DRs are shown in Fig. 7. For neonatal subjects warped to the proposed neonatal atlas, significantly better spatial consistency (p<0.05) can be observed, compared with any of the other three atlases. For 1-year-old and 2-year-old subjects, our atlas shows slightly better performance than others, although not as significant as the neonatal atlas did. The structural agreement in normalized neonates is lower than that of 1- and 2-year-old images, which may be due to the larger variances caused by insufficient image quality of the neonatal images. No significant performance difference is found between the other three control atlases. This experiment illustrates that, the 1-year-old atlas (CCHMC-Infant) does not sufficiently reflect the neonatal subjects, and the proposed neonatal atlas can significantly improve normalization consistency.

#### 3.2.2 Label Propagation

**Figure 8:**
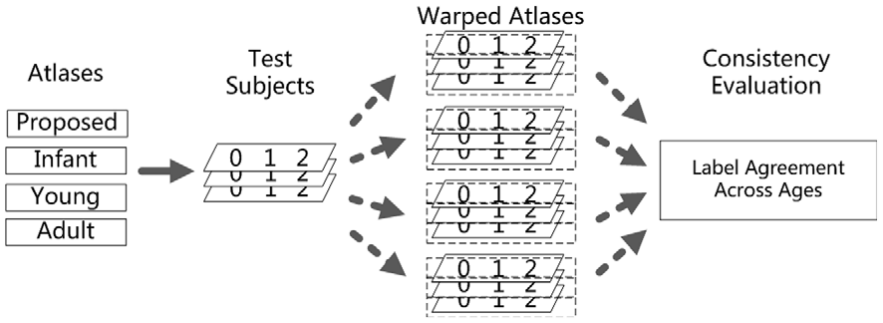
Flowchart for the label propagation experiment. The templates of atlases were aligned to the intensity images of subjects by using the normalise module in SPM5. Brain parcellation maps were propagated to subject images using the resulting deformation fields. The 116 labels were compared across the age groups for consistency evaluation.

Automated ROI delineation (or label propagation) is desired in many image analysis studies. To achieve this, usually, a template is registered to the subjects and then the anatomical parcellation map defined on the template space is warped to subjects' native spaces. If the group of subjects has a large age range, ideally, the warped brain parcellation maps should be consistently labeled on the same anatomical regions. In this experiment, we evaluated the consistency in terms of label propagation, to highlight the effectiveness of our unique longitudinal infant atlases. In particular, the templates of the 4 atlases were registered to the intensity images of the 20 test subjects by using the normalise module in SPM5, as shown in Fig. 8. For each test subject, each atlas was first warped to its respective neonatal, 1-year-old, and 2-year-old images. Specifically, for the proposed atlases, the age-matched atlas is used in registration. For the 3 control atlases, the same atlas is applied to all 3 age groups of subjects. We then compared the label overlap for each pair of the three warped parcellation maps at three different age groups of each subject, i.e., 0 vs 1, 0 vs 2, and 1 vs 2. To make the images of different age groups comparable, the longitudinal anatomical correspondence obtained in atlas construction process is utilized to warp them into the same space before comparison. Note that the CCHMC-Infant and CCHMC-Young atlases are originally not including an anatomical parcellation map. We thus construct one for each of these atlases by directly warping the AAL parcellation map to their spaces before the experiment.

**Figure 9:**
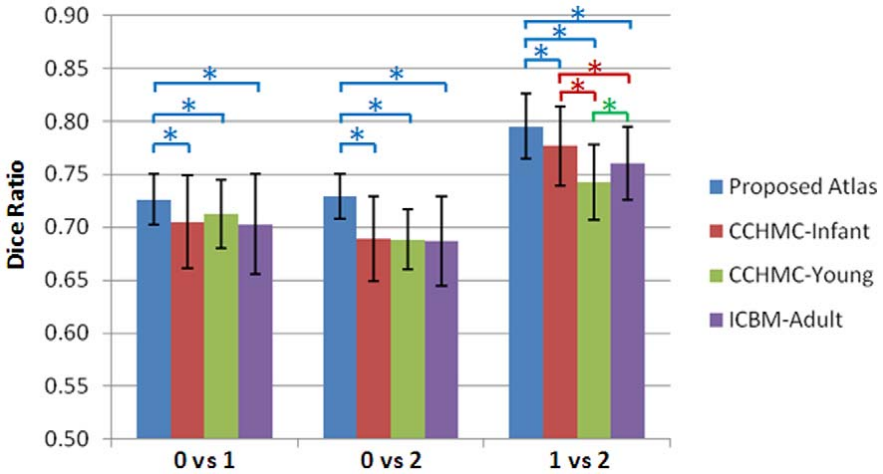
Dice ratios of the label consistency between the warped brain parcellation maps in each age group pair: 0 vs 1, 0 vs 2, and 1 vs 2. “*” represents the significant difference between groups under comparison (p<0.05).

Results are shown in Fig. 9. Our proposed atlases achieve significantly better consistency (p<0.05) than any of other three methods in all pairs. This is not surprising since that our atlas is longitudinally based and better fits the age scope of subjects; thus large age-related anatomical variances are handled better when aligning atlases to multiple ages of each subject. We can also see that the structural agreement is better achieved for the comparison 1 vs 2, which may be because the 1- and 2-year-old images are anatomically more similar to each other.

#### 3.2.3 Neonatal Segmentation

TPMs can be used to guide infant image segmentation. Atlas-based tissue segmentation is more needed for neonatal images, due to their relatively lower quality in spatial resolution, signal-to-noise ratio, and tissue contrast. For evaluation, we show only the performance of neonatal segmentation, since it is much more difficult than the segmentation of more matured subjects [28].

We manually segment 10 subjects (6 males and 4 females) from our neonatal MRI database to serve as the ground-truth. Segmentations were performed on 2 sagittal slices, 3 coronal slices, and 3 axial slices of the T2 images using the ITK-SNAP software [31] by a manual rater. For atlas-based segmentation, we employed the segmentation module distributed in SPM5 software [48]. TPMs were first warped to the native space of subjects, and segmentation was then performed by replacing the default TPMs with the warped TPMs of the 4 atlases.

**Figure 10:**
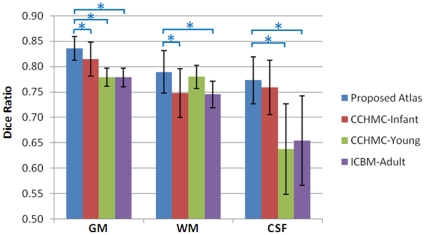
Dice ratios of segmentation consistency between manual and automated algorithms with 4 atlases. Manual segmentation was used as ground truth for comparison. GM, WM, and CSF segmentation accuracy for 10 neonatal images are averaged respectively. “*” shows the significant difference between the results obtained by the proposed atlas and other atlases (p<0.05).

The DRs of the 10 neonatal images, between automated and manual segmentations, are shown in Fig. 10. For GM, the average DR given by the proposed neonatal atlas is significantly higher than that of CCHMC-Young and ICBM-Adult; for WM, the average DR given by the proposed neonatal atlas is significantly higher than that of CCHMC-Infant and ICBM-Adult; and for CSF, the average DR given by the proposed atlas is significantly higher than that of CCHMC-Young and ICBM-Adult. These results illustrate that overall the proposed atlas yields results consistently closer to the manual segmentations.

## Discussion

We have illustrated that the proposed infant 0–1–2 atlas outperforms the 1-year-old atlas (CCHMC-Infant) in all the experiments. Additionally, using an adult atlas (ICBM-Adult) or even a pediatric atlas (CCHMC-Young) has been confirmed to compromise accuracy in analyzing infant brain images. The improved performance of our atlases mainly stems from two aspects. First, accurate tissue segmentation and subject image registration are contributive to the quality of the constructed atlas. Second, the rapid changes seen in the developing brain reinforce the importance of age-matched neonatal atlases. Our proposed publicly-available longitudinal atlases enable the selection of age-matched atlases for better guiding of tissue segmentation and structural labeling. Moreover, the longitudinal nature of our proposed 0-1-2 atlases is also critical for across-age analyses.

The AAL map was utilized for ROI definition in this paper. AAL was delineated based on anatomical characteristics, such as the main sulci, which have been shown to exist from birth and are preserved throughout the normal brain development. We have proposed two strategies to improve the consistency of AAL map propagation from the adult Colin27 brain to the infant images. First, intermediate images used as bridge images were used to reduce the possible registration errors due to large anatomical variances between AAL and infant images. Specifically, the AAL map was first propagated to the 2-year-old images which have already shown early-adult-like brain structural patterns, and then propagated to the images of neonates and 1-year-olds based on their longitudinal correspondences. Second, the AAL map was not directly registered to the group mean image. Instead, it was propagated to all 95 subjects and then fused to the final result via voxel-wise majority voting. Better image quality can be achieved for the constructed atlases by using this indirect label fusion approach [13]. By doing so, the infant AAL map constructed in this paper is anatomically consistent with the original AAL map.

The constructed brain parcellation maps are useful for many ROI–based infant studies. Besides the conventional volumetric measurement, functional correlation, and white matter fiber analysis in structural, functional and diffusion-weighted MR studies, these parcellation maps can also be used for ROI definition in the recently emerged brain connectivity studies, also called as human connectome analysis. To apply such analysis on infant subjects, a critical prerequisite is to determine the anatomical correspondences consistently. Our study, equipped with unique longitudinal dataset and an accurate label propagation method, provides the necessary brain parcellation maps for neonates, 1-year-olds, and 2-year-olds, as well as the longitudinal correspondences in between them. Network analysis on infants can thus be conducted with higher precision by avoiding directly warping the original AAL map to neonates for ROI definition.

The quality of the atlas is influenced by many factors. Age-related anatomical variance is a major factor which has been discussed in this paper. Other factors such as the number of subjects, MRI field strength, linear or nonlinear registration methods used in atlas construction, and the sharpness of the atlas also have certain impacts on the constructed atlases. However, large sample size, high MRI field strength, and joint registration and segmentation algorithm [49] are accepted as conditions for constructing a good atlas, which were all compiled in this study.

Atlases were averaged from the population in this study to represent subject-independent information in terms of intensity profile, tissue distributions, and locations of ROIs. Recently, some studies have proposed to directly use the subjects as multiple atlases [24], [27]. Multiple registrations/segmentations can be performed independently with the subject atlases and further fused into final outcome. Although computational expensive, the multiple-atlas-based methods have been demonstrated to yield promising results. In future, we plan to release our data of individual subjects to be used as multiple atlases after extensive evaluations.

WM myelination process in early brain development has also raised many research interests. However, to reflect myelination information in the atlas is a difficult task, since myelination happens rapidly in the first years of life. We currently are recruiting volunteers for a longitudinal study, in which scanning is performed at an interval of 3 months, starting from birth. This dataset would provide an opportunity to take into account myelination changes for constructing more precise infant atlases or a 4D spatial-temporal atlas as explored in [26], [50].

In summary, we have constructed the infant atlases from a unique dataset including 95 normal infant subjects with complete neonatal, 1-, and 2-year-old images. State-of-the-art techniques were employed for accurate tissue segmentation and groupwise image registration. Atlases were constructed with components of template, tissue probability maps (TPMs), and anatomical parcellation map. Longitudinal correspondence across the 3 age groups was also established to facilitate the studies with large age-range subjects. The proposed atlases were shown effective for spatial normalization of a population of infant images, as well as their anatomical labeling and tissue segmentation. We expect that the public availability of the proposed atlas and its methodology are likely to be of use to the neonatal/pediatric imaging community.
